# Mapping of Leaf Rust Resistance Loci in Two Kenyan Wheats and Development of Linked Markers

**DOI:** 10.3390/genes15050583

**Published:** 2024-05-03

**Authors:** Davinder Singh, Peace Kankwatsa, Karanjeet S. Sandhu, Urmil K. Bansal, Kerrie L. Forrest, Robert F. Park

**Affiliations:** 1 Plant Breeding Institute, School of Life and Environmental Sciences, University of Sydney, Narellan, NSW 2567, Australia; karanjeet.sandhu@sydney.edu.au (K.S.S.); urmil.bansal@sydney.edu.au (U.K.B.); robert.park@sydney.edu.au (R.F.P.); 2 National Agricultural Research Organization, MbaZARDI, Mbarara City P.O. Box 389, Uganda; kankwatsap@gmail.com; 3 Agriculture Victoria, AgriBio, Centre for AgriBioscience, Bundoora, VIC 3083, Australia; kerrie.forrest@agriculture.vic.gov.au

**Keywords:** wheat (*Triticum aestivum*), leaf rust, *Puccinia triticina*, resistance, breeding, mapping, KASP markers

## Abstract

Leaf rust caused by the pathogen *Puccinia triticina* (*Pt*) is a destructive fungal disease of wheat that occurs in almost all wheat-growing areas across the globe. Genetic resistance has proven to be the best solution to mitigate the disease. Wheat breeders are continuously seeking new diversified and durable sources of resistance to use in developing new varieties. We developed recombinant inbred line (RIL) populations from two leaf rust-resistant genotypes (Kenya Kudu and AUS12568) introduced from Kenya to identify and characterize resistance to *Pt* and to develop markers linked closely to the resistance that was found. Our studies detected four QTL conferring adult plant resistance (APR) to leaf rust. Two of these loci are associated with known genes, *Lr46* and *Lr68*, residing on chromosomes 1B and 7B, respectively. The remaining two, *QLrKK_2B* and *QLrAus12568_5A*, contributed by Kenya Kudu and AUS12568 respectively, are putatively new loci for *Pt* resistance. Both *QLrKK_2B* and *QLrAus12568_5A* were found to interact additively with *Lr46* in significantly reducing the disease severity at adult plant growth stages in the field. We further developed a suite of six closely linked markers within the *QLrAus12568_5A* locus and four within the *QLrKK_2B* region. Among these, markers *sunKASP_522* and *sunKASP_524*, flanking *QLrAus12568_5A*, and *sunKASP_536*, distal to *QLrKK_2B*, were identified as the most closely linked and reliable for marker-assisted selection. The markers were validated on a selection of 64 Australian wheat varieties and found to be polymorphic and robust, allowing for clear allelic discrimination. The identified new loci and linked molecular markers will enable rapid adoption by breeders in developing wheat varieties carrying diversified and durable resistance to leaf rust.

## 1. Introduction

Wheat accounts for about 20% of the total calories and protein consumed annually by the planet’s 7.9 billion people [1], providing more nourishment for humankind than any other food source. Future global wheat production is however impeded by factors like population growth, scarcity of suitable farmland, a decline in the genetic diversity of commercial cultivars, and various abiotic and biotic stresses. The biotic threats (primarily diseases, insects, animals, and weeds) can collectively result in significant damage accounting for 10–50% of wheat crop losses worldwide [2]. Amongst various wheat diseases, those caused by fungi pathogens including the three rusts (leaf rust, stem rust and stripe rust) are considered the most significant in hindering the global wheat supply. 

Of the three rust diseases of wheat, leaf rust caused by pathogen *Pt* is the most widespread, causing significant production losses worldwide by affecting kernel weight and number, and crop biomass [3,4]. Although fungicides have proven useful in controlling leaf rust in some parts of the world, genetic resistance remains the foundation of integrated disease control. Deployment of varieties with better levels of disease resistance is not only economically viable, but also reduces the need for fungicide applications, thereby protecting the efficacy of fungicides and benefiting the environment. 

Breeding for resistance to *Pt* is challenging because of the continuous evolution of the pathogen resulting in new variants (pathotypes) that can overcome resistance. This necessitates the ongoing discovery of new sources of resistance, especially those associated with durable resistance. In a broad sense, cereal rust resistance including resistance to *Pt* can be classified as either qualitative or quantitative, the former being typically controlled by race specific genes (R genes) of major effect, and the latter based on multiple genes each with a minor/partial effect. The resistance contributed by single major R genes is generally expressed at all plant growth stages and is hence often referred to as all stage resistance (ASR). In contrast, minor/partial resistance is evident only at post-seedling growth stages and is therefore referred to as adult plant resistance (APR). Due to the incomplete level of protection conferred by minor APR genes, combinations of multiple APR genes are often needed to achieve acceptable to good levels of resistance. 

To achieve durable resistance, it is crucial to recognize and separate the effects of know major ASR genes from minor APR genes. Equally important is the characterization and mapping of newly discovered resistance genes. This ensures the diversity of effective resistance and enables efficient incorporation in germplasm enhancement programs. Early genetic mapping studies prior to 1990s were based mainly on classical genetic and cytogenetic techniques, like linkage and recombination analysis, monosomic analyses, etc. [5]. However, these techniques were time consuming and largely confined to chromosome location of resistance genes. With the advent of high-throughput molecular marker technologies based on early generation platforms such as restriction fragment length polymorphism (RFLPs), random amplified polymorphic DNA (RAPDs), amplified fragment length polymorphism (AFLPs), followed by simple sequence repeats (SSRs), single nucleotide polymorphisms (SNPs), and the availability of high-density genetic maps in wheat [6,7], rust resistance gene mapping efforts improved over time. The development of SNP gene-chip technology and the recent publication of the whole genome/Pan-genome sequencing and annotating in wheat (Chinese Spring RefSeq v2.1 [8,9]) has further improved the efficiency of fine mapping, gene cloning, and the development of diagnostic markers for marker-assisted selection (MAS) and pyramiding of resistance genes. 

Diverse wheat germplasm conserved in national and international collections is a valuable resource for identifying new sources of resistance to biotic stresses including *Pt*. Kankwatsa et al. [10] assembled an African wheat collection spanning 10 countries, identified potentially new ASR and APR to leaf rust in several genotypes, and recommended their further characterization and mapping. We developed recombinant inbred line (RIL) populations from two genotypes (Kenya Kudu and AUS12568) that had high levels of resistance to leaf rust with the aims of (i) identifying, characterizing, and mapping APR to *Pt*, (ii) understanding gene interaction among the resistances identified, and (iii) developing markers linked closely to the resistances found.

## 2. Materials and Methods

Plant and pathogen resources: The leaf rust-resistant genotypes Kenya Kudu (Pedigree: Kenya 131/Kenya 184 P) and AUS12568 (Pedigree: unknown) investigated in this study were introduced to Australia by the Australian Grain Gene (AGG) bank (Horsham, VIC, Australia) from Kenya. Kankwatsa et al. [10] developed F_3_ populations from both genotypes by crossing each to a leaf rust-susceptible line AWDH161 (developed and maintained at the Plant Breeding Institute (PBI) Cobbitty, NSW, Australia). We developed RILs from each of the F_3_ populations (Kenya Kudu/AWDH161, *n* = 106, AUS12568/AWDH161, *n* = 108) by advancing these populations to F_7_ using single-seed descent followed by single-plant bulking of each F_7:8_ line. The parental lines were tested in the greenhouse with four Australian *Pt* pathotypes [76-1,3,5,7,9,10,12,13 + Lr37 = culture number 630, 104-1,2,3,(6),(7),11,13 = 547, 104-1,3,4,6,7,8,10,12 + Lr37 = 634; 104-2,3,6,(7) = 231]. The pathotype nomenclature is described in the review by Park [11]. An admixture of the same four pathotypes was also used in the field to generate rust epidemics for adult plant screening. These pathotypes were chosen because they are either common in Australia and/or are virulent on many of the ASR genes present in global and Australian wheat germplasm (including *Lr1*, *Lr3a*, *Lr3ka*, *Lr10, Lr12, Lr13*, *Lr14a*, *Lr15*, *Lr16*, *Lr17a*, *Lr17b*, *Lr20*, *Lr23*, *Lr24, Lr26*, *Lr27+31*, *Lr28*, *Lr37* and *Lr73*) and are broad spectrum representations of the current Australian leaf rust pathogen population.

Sowing and disease development procedures: The parental genotypes and a standard Australian set of leaf rust differentials [12] were sown as four clumps (8–10 seeds/clump) per 9 cm diameter pot. Seedlings were raised at 20–22 °C in greenhouse growth rooms with regular watering and a weekly application of a nitrogenous fertilizer (Aquasol^R^) at a rate of 25 g per 10 L of water for 100 pots. Leaf rust inoculations were performed typically on 10-day old seedlings when they reached the 1–1.5 leaf stage. Urediniospores suspended in a non-phytotoxic light mineral oil (Isopar L, Sydney, NSW, Australia; 10 mg of spores per 10 mL per 200 pots) were atomized evenly over seedlings using a hydrocarbon propellant pressure pack. The inoculated seedlings were incubated for 18–24 h in a dark chamber at ambient temperatures where mist was generated by ultrasonic humidifiers. After incubation, infected seedlings were transferred to naturally lit temperature and irrigation-controlled microclimate chambers maintained at 23 °C for further disease development. 

The parental lines and the RIL populations were sown in the field at Cobbitty in June 2019. Approximately 30 seeds of each line were sown in 0.75 cm rows at 0.25 cm intervals using a single-row drill HEGE 90 planter mounted onto a tractor. A spreader row comprising of leaf rust-susceptible wheat genotypes (Morocco, Naparoo, Sonora and Yitpi) was sown after every five rows of test lines to ensure high inoculum build-up and uniform disease development. The resistant and susceptible parental lines were included at the beginning, middle and end of each RIL population as controls. The field epidemics were produced by misting urediniospores suspended in mineral oil (Isopar^TM^ L, Union Petrochemical Public Company Limited, Bangkok, Thailand, 10 mg of spores per 10 L per 1000 m^2^) over the disease spreader rows using an ultra-low-volume applicator (Microfit^®^, Micron Sprayer Ltd., Bromyard, UK) on at least two clear afternoons when there was a high likelihood of overnight dew formation. The conditions for pathogen growth from inoculations to disease development (over 2 months) were favorable with optimal rainfall (200 mm), controlled springer irrigation (20 mm per week), and temperature ranging from 10 to 26 °C.

Phenotyping and disease assessment: The rust response on seedlings in greenhouses was recorded using the modified Stakman scale [13], where ‘0’ represents no visible uredinia, ‘;’ represents hypersensitive flecks, 1–4 represents increasing sporulation in the pustules and ‘X’ represents the variable size pustules on the same leaf. Infection types of 3+ to 4 were considered compatible or high (susceptible) and 0 to 3 incompatible or low (resistant). Adult plant leaf rust response was assessed in the field based on a 1–9 scale described by Sandhu et al. [14]. The original scale was developed for stripe rust resistance and we modified it to leaf rust resistance, where 1 is very resistant (0% leaf area affected with no visible infection), 2 is resistant (~10% leaf area affected with very restricted uredinia), 3 is resistant to moderately resistant (~20% leaf area affected with very small uredinia), 4 is moderately resistant (~30% leaf area affected with small to medium uredinia), 5 is moderately resistant to moderately susceptible (~40% leaf area affected with medium uredinia), 6 is moderately susceptible (~50% leaf area affected with medium to large uredinia), 7 is moderately susceptible (~70% leaf area affected with medium to large uredinia), 8 is susceptible (~80% leaf area affected with large uredinia) and 9 is very susceptible (>90% leaf area affected with large uredinia and abundant sporulation). 

The lines within each population were planted as single 1 m rows. To ensure the uniformity of pathogen infection, a mixture of susceptible cultivars was sown as a disease spreader after every 5 lines. Leaf rust response variation was assessed post-anthesis when the susceptible parent AWDH161 (planted after every 20 lines) exhibited disease symptoms corresponding to pathogenic response ‘8’ to ‘9’ on a 1–9 scale. Disease assessments were made twice at a 7-day interval. An average was taken of two readings for calculating disease score (DS) for each line. 

Genotyping and molecular mapping: Genomic DNA from the two RIL populations and their respective parents was extracted from young leaf tissue (single plant per genotype) using a modified CTAB method [15] and further quantified before genotyping. Both mapping populations were genotyped by GrainDataGen™ at Agriculture Victoria using a targeted genotype via sequencing (tGBS) assay for 11 k exome SNPs. Samples were analyzed using a custom bioinformatics pipeline that processes sample reads from the tGBS assay to generate genotype calls for polymorphic loci. The observed parental genotype calls were used to recode the sample genotypes for genetic map construction according to parental origin. When the observed parental alleles did not segregate in the mapping population samples, or did not show expected Mendelian inheritance, the parental genotype was inferred. This enabled all polymorphic markers to be considered for integration into the genetic map. Markers were ordered by position in the genome assembly, then haplotypes were assessed to assign markers to chromosome. After the construction of genetic linkage maps, the resistant and susceptible phenotypes of RILs were converted into genotypes A and B, and data were incorporated into the developed map for locating/mapping the resistance on the genome. Markers linked to *Lr34* (csLV34, [16], *Lr46* (*csLV46G22*, [17], and *Lr67*, (TM4, [18]) were used to determine the presence/absence of these loci in the test genotypes.

QTL analyses: The Composite Interval Mapping (CIM) analyses were performed for the detection of QTL for rust response using QTL Cartographer [19]. QTL were identified for both diseases using mean data for two seasons. The trait threshold Logarithm of the Odds (LOD) values were calculated at a manually set number of permutations and the significance level. In this study, 1000 permutations at *p* = 0.01 were used. QTL with LOD scores > 3.0 were considered significant. Final linkage map figures were prepared using MapChart software (version: 2.32) [20].

Development and application of KASP markers: Linked SNPs were converted to Kompetitive Allele-Specific PCR (KASP) markers using the software Polymarker (http://www.polymarker.info, accessed on 25 April 2022). The KASP markers developed were tested on the entire RIL populations and further validated on a set of 64 Australian varieties and 2 susceptible controls, Morocco and AvocetS (Supplementary Table S1). PCR amplifications were performed in 8 μL reaction volumes containing 90 ng genomic DNA, 0.11 μL of KASP primer mix, 4 μL PACE mix (3CrBioscience, Harlow, UK) and 0.89 μL of milliQ water [21]. The 64 varieties represent three wheat cropping zones of Australia (North, South and West). The rust resistance phenotype of these varieties (Supplementary Table S1) was obtained from various Australian Cereal Rust Control Program circulars and aligned against each marker. Marker *sunKASP_522* A and *sunKASP_536* amplified the ‘A’ allele in the resistant parental stock AUS12568 and Kenya Kudu, respectively, and the alternate ‘B’ allele in the susceptible parent AWDH161. The test varieties carrying the ‘A’ allele for respective markers were considered to carry resistant alleles.

## 3. Results

### 3.1. Phenotypic Assessment of Parental Lines and RILs

**Figure 1:**
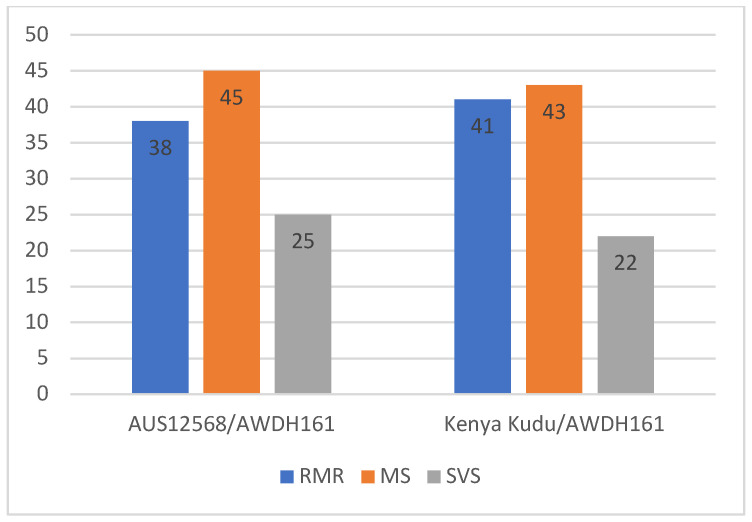
Genetic diversity and distribution of adult plant leaf rust resistance response in two mapping populations (RMR = resistant to moderately resistant, MS = moderate susceptible, SVS = susceptible to very susceptible).

Parental lines Kenya Kudu, AUS12568 and AWDH161 were susceptible to all four *Pt* pathotypes [76-1,3,5,7,9,10,12,13 + Lr37, 104-1,2,3,(6),(7),11,13, 104-1,3,4,6,7,8,10,12 + Lr37, and 104-2,3,6,(7)] at seedling growth stages. When tested in the field as adult plants using an admixture of the same four pathotypes, AUS12568 and Kenya Kudu were highly resistant and AWDH161 was susceptible (Table 1). This confirmed that the resistance in Kenya Kudu and AUS12568 to the four pathotypes used is conferred by APR and not ASR. When tested in the field at adult plant growth stages, the two RIL populations segregated for susceptibility and continuous variation in resistance ranging from R to MS was observed (Table 2, Figure 1). The lines with disease scores 8–9 were classified as susceptible (S) and the remaining as resistant (R). Genetic analysis (based on R versus S classification) revealed a strong fit for two-gene inheritance (3R:1S ratio) in both populations [*p* > 0.31 (Kenya Kudu/AWDH161) and *p* > 0.48 (AUS12568/AWDH161) at 1 df, Table 2)].

### 3.2. Mapping of Resistance

Two QTL conferring resistance to leaf rust were detected on chromosomes 1B and 2B in the population Kenya Kudu/AWDH161, both contributed by the parent Kenya Kudu (Table 3). The 1B QTL (*QLrKK_1B*) corresponded to the location of *Lr46* (based on the search of *Lr46* closely linked markers against the reference genome of common wheat ‘Chinese Spring’ v1.1 (CSRGv1.1)) and explained 41.9% of phenotypic variance (PVE%). Markers associated with the 2B QTL (*QLrKK_2B*) were located at a physical position of 697.7–707.10 Mb in CSRGv1.1 and explained relatively less PVE (18.9%) but still had a major effect in lowering the disease response. For the population AUS12568/AWDH161, three QTL were detected that were significantly associated to markers on chromosomes 1B, 5A, and 7B contributed by parent AUS12568 (Table 3). The 1B QTL (*QLrAus12568_1B*) contributed 19.9% PVE and again corresponded to gene *Lr46*. The 5A QTL (*QLrAus12568_5A*, PVE = 15.2%) and 7B QTL (*QLrAus12568_7B*, PVE = 17.3%) were positioned at 560.6–594.1 Mbp and 29.5–46.3 Mbp, respectively, using CSRGv1.1. To further confirm the presence of *Lr46* in both populations, the parents and populations were tested with *Lr46*-linked KASP marker *csLV46G22*. Both resistant parents were positive for the *Lr46* marker and populations segregated for *Lr46*/*lr46* locus [*χ*^2^1:1 = 0.76, *p* > 0.38 (Kenya Kudu/AWDH161) and = 1.35, *p* > 0.24 (AUS12568/AWDH161) at 1 df].

### 3.3. Development and Validation of KASP Markers

**Figure 2:**
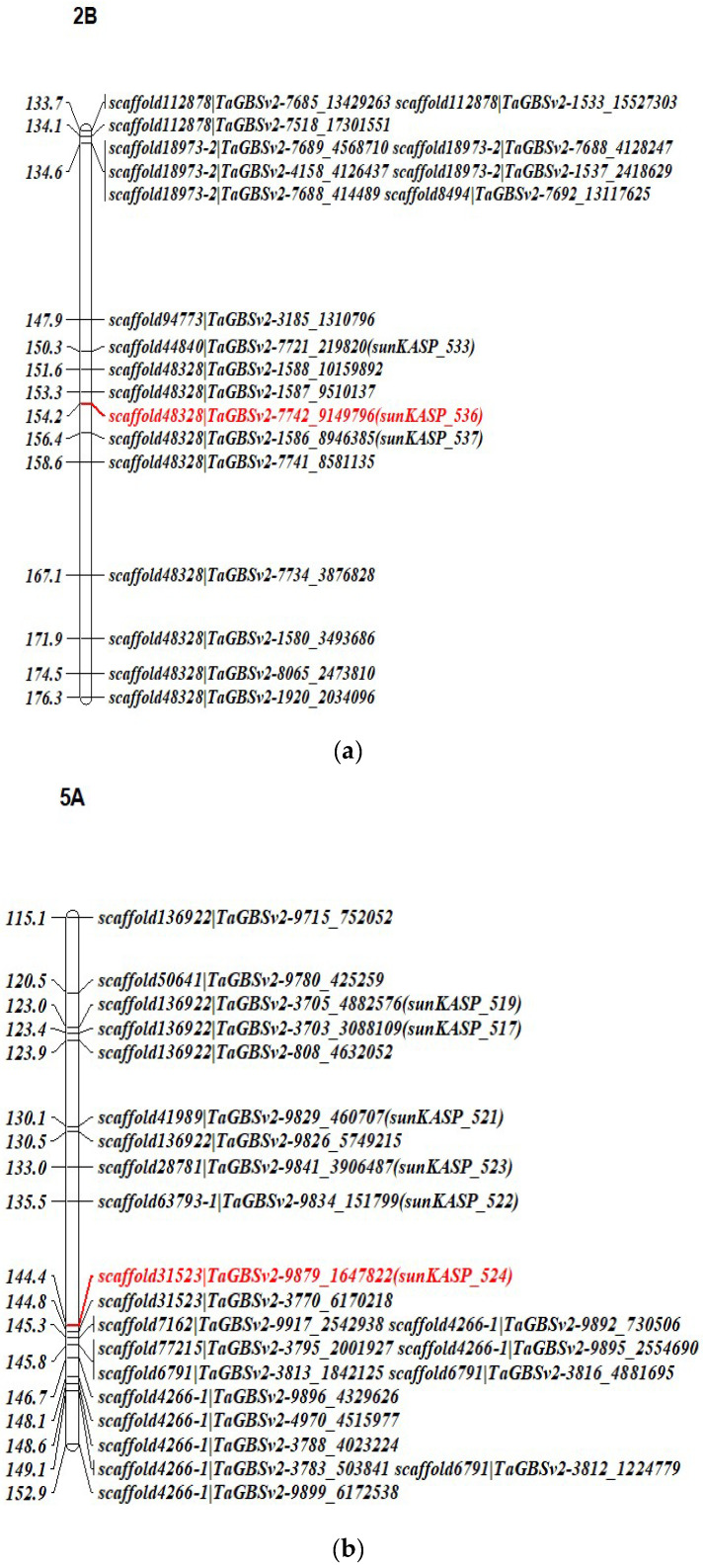
Genetic linkage map of chromosome 2B (**a**) and 5A (**b**) showing the position of KASP markers in Kenya Kudu/AWDH161 and Aus12568/AWDH1 RIL populations (position of scaffold and closely linked KASP marker to *QLrKK_2B* and *QLrAus12568_5A* highlighted in red).

Six markers associated with the linked SNPs in the *QLrKK_2B* region (697.7–707.10 Mb) from the population Kenya Kudu/AWDH161 were converted into KASP assays. Of these six markers, only three (*sunKASP_533*, *sunKASP_536* and *sunKASP_537*) were polymorphic in the parents, among which *sunKASP_536* (scaffold48328|TaGBSv2-7742_9149796, Figure 2a) produced the best clusters and was found to be polymorphic on a panel of 64 Australian wheat varieties. Of the sixty-four varieties genotyped with *sunKASP-36*, only two lines (Sunmax and Sunzell) carried the *QLrKK_2B* allele (Supplementary Table S1) and both were found to be resistant in previous studies. Similarly, six polymorphic KASP markers (*sunKASP_517*, *sunKASP_519*, *sunKASP_521*, *sunKASP_522*, *sunKASP_523* and *sunKASP_524*) were developed within *QLrAus12568_5A* (560.6–594.1 Mb), of which *sunKASP_522* (scaffold63793-1|TaGBSv2-9834_1517999, Figure 2b) and *sunKASP_524* (scaffold31523|TaGBSv2-9879_1647822, Figure 2b) produced the best well-defined allelic discrimination. Marker genotyping of the 64 Australian wheat varieties with *sunKASP_524* revealed the presence of the *QLrAus12568_5A* allele in four varieties (Dart, Bonnie Rock, King Rock and Shield; Supplementary Table S1) and all four showed resistant phenotypes. As expected, both susceptible controls (Morocco and Avocet S) showed allele amplification corresponding to the susceptible parent AWDH161. The primer sequences and positions of the developed KASPs are presented in Table 4 and Figure 2a,b.

### 3.4. Assessment of Interaction among Detected APR Loci

Both populations were tested with marker *csLV46G22* linked to *Lr46*, and *KASP_536* and *KASP_524* developed for *QLrKK_2B* and *QLrAus12568_5A* APR loci detected in Kenya Kudu/AWDH161 and AUS12568/AWDH161 populations, respectively. Based on joint marker analysis, each population was partitioned into four genotypic classes and disease scores for each class were averaged (Table 5). The lines carrying resistance loci (singly or in combination) had a significant reduction in average disease severity (DS) when compared with RILs lacking these resistance loci in both populations. For AUS12568/AWDH161, RILs carrying *Lr46* and *LrAus12568* singly did not differ significantly from each other for mean DS, but in combination (*Lr46* + *LrAus1268_5A*) resulted in a significant reduction in average DS. This implies that *Lr46* and *LrAUS12568* interact additively. For the population Kenya Kudu/AWDH161, no significant differences were found between lines carrying *Lr46* and *QLrKK_2B* singly. The lines carrying *LrKK_2B* and *Lr46* in combination did not significantly differ from lines carrying *LrKK_2B* singly but differed from lines carrying *Lr46* singly.

## 4. Discussion

The efforts of researchers across the globe have led to the identification, characterization, and mapping of over 80 catalogued leaf rust resistance genes, the most recent of which being *Lr82* [22]. In addition, numerous quantitative trait loci (QTL) and marker trait associations (MTA) for resistance to leaf rust have also been reported [23]. Despite the availability of such an enormous gene pool, the impact of these genes in resistance breeding has been constrained because many have proven non-durable and/or are associated with undesirable linkage drag (yield penalties, etc.) and/or lack of suitable markers available for MAS. This highlights the need to identify sources that are more durable, carry out their genetic characterization, and obtain a greater understanding for the deployment of wider diversity and development of robust PCR-based markers that can be efficiently used for MAS.

In the present study, we identified three loci conferring APR to leaf rust of which one corresponds to the known pleiotropic APR locus *Lr46*/*Yr29*/*Sr58* on chromosome 1B and two undetermined loci located on chromosomes 2B (contributed by cv. Kenya Kudu, tentatively designated *QLrKK_2B*) and 5A (contributed by line AUS12568, designated *QLrAus12568_5A*) conferring APR. To date, only 13 genes have been catalogued in wheat that confer APR to *Pt* (*viz*. *Lr12*, *Lr22a*, *Lr22b*, *Lr34*, *Lr35*, *Lr46*, *Lr48*, *Lr49*, *Lr67*, *Lr68*, *Lr74*, *Lr75* and *Lr77*) [23], of which five confer a hypersensitive major effect phenotype (*Lr12*, *Lr22a*, *Lr22b*, *Lr48* and *Lr49*) and seven of which confer a minor phenotypic effect (*Lr34*, *Lr46*, *Lr67*, *Lr68*, *Lr74*, *Lr75* and *Lr77*). 

Wheat chromosome 2B also harbors several known and unknown *Lr*/*QLr* loci including the widely deployed/common ASR genes *Lr13*, *Lr16*, *Lr23*, *Lr50*, *Lr58* and *Lr73*. Multi-pathotype tests in our study indicated that none of these genes are present in Kenya Kudu, and all can therefore be excluded as being candidates for *QLrKK_2B*. The APR genes *Lr35* and *Lr48* are also located on 2B but are located distantly from *QLrKK_2B* (>300 Mb) based on consensus locations of leaf rust loci estimated by Ren et al. [24] using positions of associated molecular markers in the Chinese Spring reference genome. The markers *Xbcd260* and *Xgwm429b* closely linked to *Lr35* and *Lr48*, respectively, were absent in Kenya Kudu, providing further evidence of the genetic independence of *QLrKK_2B* from *Lr35* and *Lr48*. At least five QTL or MTAs conferring resistance to *Pt* have also been reported on the short arm of chromosome 2B [24], contributed by the varieties Saragolla [25], Attila [26], Catbird [27] and Capo [28], but it is highly unlikely that any of these QTL correspond to *QLrKK_2B* because of its location on the 2B long arm. An APR QTL *QLr.dms.2B.2* was reported on the long arm of 2B in a GWAS study [29] at 760.3 Mb (associated with marker *Excalibur_c62234_105*) on a comparative Chinese Spring reference genome map of Ren et al. [24], but is still largely distant to *QLrKK_2B* (697.7–707.10 Mb). Recently, Bariana et al. [22] mapped a new seedling resistance loci *Lr82* on chromosome 2B. However, the presence of *Lr82* in Kenya Kudu can be ruled out because it showed a susceptible response to two *Lr82*-avirulent pathotypes (104-1,3,4,6,8,10,12 + Lr37 and 76-1,3,5,7,9,10,12,13 + Lr37; Table 1) used in this investigation. Based on this comparative position analysis of *QLrKK_2B* with previously detected *Lr*/*QLr*, it can be assumed that the *QLrKK_2B* is novel. 

The locus *QLrAus12568_5A* detected in AUS12568 also appears new as no other known catalogued *Lr* gene has been reported on chromosome 5A. Nevertheless, at least six QTL or MTAs conferring resistance to *Pt* have been reported previously on 5A of which the one reported from European winter wheat Beaver [4], and MTAs from three independent GWAS spring and winter wheat panels [30,31,32] are vastly distant from the 5A QTL detected in this study. Two other QTL reported in cv Toropi-6 at 580.5 Mb [33] and line SW8588 at 580.9 Mb [34] are, however, in the vicinity of the 5A QTL detected in this study at 560 to 594 Mb. Synonymy among these three QTL cannot be ruled out and an allelism test is recommended. The QTL detected on chromosome 7BL is possibly related to the known APR gene *Lr68* based on further genotyping of parent AUS12568 and several random lines selected from AUS12568/AWDH161 with the marker closely linked to *Lr68* [35]. Nevertheless, the reported locations of *QLrAUS12568_7B* and *Lr68* are very different.

Both Kenya Kudu and AUS12568 investigated in this study were found to carry *Lr46*, a gene that has been widely deployed in global wheat germplasm because of its association with durable APR and its interaction/additivity with other APR genes [4,36]. Examining the effects of *Lr46* with and without *QLrKK_2B* and *QLrAus12568_5A*, we found that *Lr46* significantly interacts additively with *QLrAus12568_5A* in reducing disease severity at adult plant stages in the field. The *QLrKK_2B* also showed apparent additivity, but this was not statistically significant. The gene additivity results suggest that both loci, in particular *QLrAus12568_5A*, can serve as good leaf rust APR donors for gene pyramiding and achieving durable resistance.

The statistical analysis performed in this study is based on single-field testing and hence may compromise the statistical power of mapping. Although our mapping results provided superior resolution in detecting genomic locations, we recommend that its statistical power should be further validated by additional multi-environment testing. Further, APR to rusts is often sensitive to environment and genotype–environment interactions, in addition to the structure of the pathogen population. The disease resistance may be affected by variables such as soil type, temperature, rainfall and therefore the disease severity associated with each QTL may fluctuate under different environmental conditions. 

Six markers closely linked to *QLrAus12568_5A* and four to *QLrKK_2B* were developed, of which *sunKASP_522* and *sunKASP_524* flanking the 5A locus and *sunKASP_536* (distal to *QLrKK_2B*) were the most closely linked and suitable for MAS. These markers when validated on a set of 64 Australian varieties were found to be polymorphic and robust with clear allelic discrimination. The identified markers (sequence information provided in Table 4) will be valuable to breeders for developing varieties with diversified resistance and long-term protection against leaf rust.

## Figures and Tables

**Table 1:** Infection-type seedling greenhouse response and adult plant field disease responses of three wheat genotypes to *Pt*.

Genotype	Infection Type Response				Disease Response
	Greenhouse				Field
	Pt. 1 *	Pt. 2	Pt. 3	Pt. 4	Pts. 1 + 2 + 3 + 4
AUS12568	3+	3+	3+	3+	5–10 RMR
Kenya Kudu	3+	33 + C	3+	3+	10 MR
AWDH161	3+	3+	3+	3+	90–100 S

* Pt. (pathotype) 1 = 76-1,3,5,7,9,10,12,13 + Lr37; Pt. 2 = 104-1,2,3,(6),(7),11,13; Pt. 3 = 104-1,3,4,6,7,8,10,12 + Lr37; Pt. 4 =104-2,3,6,(7).

**Table 2:** Distribution of leaf rust response categories and Chi-squared analyses of two RIL populations when tested for response to *Pt* in the field at the adult plant growth stage (Z59).

RIL Population	Field Disease Response Category			Segregation	Genetic Ratio	*χ* ^2^	*p*
	RMR	MS	SVS	R:S ^a^		(R:S)	
A12568/AWDH161	38	45	25	83:25	3:1	0.492	0.483
Kenya Kudu/AWDH161	41	43	22	84:22	3:1	1.019	0.310

RMR = Resistant to moderately resistant; MS = moderately susceptible; SVS = susceptible to very susceptible. ^a^ R = RMR + MS; S = SVS. *p* 0.05 = 3.84 at 1 df; *p* 0.01 = 6.64 at 1 df.

**Table 3:** Summary of the QTL detected for resistance to *Pt* in two RIL populations.

Population	QTL/Chr	Left Marker	Right Marker	LOD	PVE (%)	Contributing Parent	Position in CS Physical Map (bp)	
							Left Marker	Right Marker
AUS12568/AWDH161	*QLrKK_1B*	scaffold95194|TaGBSv2-6835_494429	scaffold95194|TaGBSv2-734_2005054	5.6	19.9	AUS12568	668,762,861	670,273,486
	*QLrKK_5A*	scaffold63793-1|TaGBSv2-9834_151799	scaffold31523|TaGBSv2-9879_1647822	5.5	15.2	AUS12568	560,600,410	594,143,849
	*QLrKK_7B*	scaffold42040-2|TaGBSv2-5623_4726145	scaffold67584|TaGBSv2-5649_9487071	3.7	17.3	AUS12568	29,588,094	46,347,338
Kenya Kudu/AWDH161	*QLrKK_1B*	scaffold48390|TaGBSv2-6823_159757	scaffold95194|TaGBSv2-6835_494429	9.1	41.9	Kenya Kudu	661,632,366	668,762,861
	*QLrKK_2B*	scaffold94773|TaGBSv2-3185_1310796	scaffold44840|TaGBSv2-7721_219820	3.9	18.9	Kenya Kudu	697,784,429	707,968,413

**Table 4:** Primer sequences for KASP markers developed for *QLrKK_2B* and *QLrAus12568_5A*.

LrQTL	Marker Name	Allele 1	Allele 2	Common
*QLrKK_2B*	*sunKASP_536*	ttgcaccattcttatatctggaatT	ttgcaccattcttatatctggaatC	actgtcagCtcattgccttca
*QLrAus12568_5A*	*sunKASP_522*	gttggatgagagctacacacC	gttggatgagagctacacacT	catcgccAgtccagatggag
*QLrAus12568_5A*	*sunKASP_524*	tgatcagtgtggcatgacaG	tgatcagtgtggcatgacaA	aacttccatgaagctgctagt

**Table 5:** Interaction of leaf rust resistance loci *QLrKK_2B* and *QLrAus12568_5A* with *Lr46* in two RIL populations.

Population	Locus	No of Lines	Average DS *
Kenya Kudu/AWDH161	*LrKK_2B*	21	4.18 ^AB^
	*Lr46*	20	5.4 ^A^
	*LrKK_2B* + *Lr46*	36	3.73 ^B^
	None	17	7.44 ^C^
	*lsd*		1.23
AUS12568/AWDH161	*LrAus12568_5A*	21	4.54 ^A^
	*Lr46*	27	5.16 ^A^
	*LrAus12568_5A* + *Lr46*	20	3 ^B^
	None	38	6.62 ^C^
	*lsd*		1.31

*lsd* = least significant differences. * within a column, average DS (disease severity) followed by different letters differ significantly.

## Data Availability

All data are given in the manuscript. Publicly available statistical tools are used in this study.

## Supplementary Materials

The following supporting information can be downloaded at: https://www.mdpi.com/article/10.3390/genes15050583/s1, Table S1: Genotyping of 64 Australian wheat varieties using markers linked to *QLrAus12568_5A* and *QLrKK_2B* and association with field leaf rust ratings.

## References

[1] FAOSTAT Statistics Database: Food Balance Sheets. http://www.fao.org/faostat/en/#data/FBS.

[2] Chai Y., Senay S., Horvath D., Pardey P. (2022). Multi-peril pathogen risks to global wheat production: A probabilistic loss and investment assessment. Front. Plant Sci..

[3] Herrera-Foessel S.A., Singh R.P., Huerta-Espino J., Crossa J., Jin Y., Djurle A. (2006). Effect of leaf rust on grain yield and yield traits of durum wheats with race-specific and slow rusting resistance to leaf rust. Plant Dis..

[4] Singh D., Simmonds J., Park R.F., Bariana H., Snape J. (2009). Inheritance and QTL mapping of leaf rust resistance in the European winter wheat cultivar ‘Beaver’. Euphytica.

[5] Singh D., Park R.F., Bariana H.S., McIntosh R.A. (2001). Cytogenetic studies in wheat XIX: Chromosome location and linkage studies of a gene for leaf rust resistance in the Australian cultivar Harrier. Plant Breed..

[6] Cavanagh C.R., Chao S., Wang S., Huang B.E., Stephen S., Kiani S., Forrest K., Saintenac C., Brown-Guedira G.L., Akhunova A. (2013). Genome-wide comparative diversity uncovers multiple targets of selection for improvement in hexaploid wheat landraces and cultivars. Proc. Natl. Acad. Sci. USA.

[7] Somers D.J., Isaac P., Edwards K. (2004). A high-density microsatellite consensus map for bread wheat (*Triticum aestivum* L.). Theor. Appl. Genet..

[8] International Wheat Genome Sequencing Consortium (IWGSC) (2018). Shifting the limits in wheat research and breeding using a fully annotated reference genome. Science.

[9] Zhu T., Wang L., Rimbert H., Rodriguez J.C., Deal K.R., De Oliveira R., Choulet F., Keeble-Gagnère G., Tibbits J., Rogers J. (2021). Optical maps refine the bread wheat *Triticum aestivum* cv. Chinese Spring genome assembly. Plant J..

[10] Kwankwaso P., Park R.F., Singh D. (2019). African wheat germplasm—A valuable resource for resistance to rust diseases. Plant Pathol..

[11] Park R. (2008). Breeding cereals for rust resistance in Australia. Plant Pathol..

[12] McIntosh R., Wellings C.R., Park R.F. (1995). Wheat Rusts: An Atlas of Resistance Genes.

[13] Stakman E.C., Stewart D.M., Loegering W.Q. (1962). Identification of Physiologic Races of Puccinia graminis var. tritici.

[14] Sandhu K., Singh D., Park R.F. (2021). A pictorial disease assessment scale for assessing wheat stripe rust at adult plant growth stage. Aust. Plant Pathol..

[15] Bansal U.K., Kazi A.G., Singh B., Hare R., Bariana H.S. (2014). Mapping of durable stripe rust resistance in a durum wheat cultivar Wollaroi. Mol. Breed..

[16] Lagudah E.S., McFadden H., Singh R.P., Huerta-Espino J., Bariana H.S., Spielmeyer W. (2006). Molecular genetic characterisation of the *Lr34*/*Yr18* slow rusting resistance gene region in wheat. Theor. Appl. Genet..

[17] Ren Y., Singh R.P., Basnet B.R., Lan C.X., Huerta-Espino J., Lagudah E.S., Ponce-Molina L.J. (2017). Identification and mapping of adult plant resistance loci to leaf rust and stripe rust in common wheat cultivar Kundan. Plant Dis..

[18] Moore J.W., Herrera-Foessel S., Lan C., Schnippenkoetter W., Ayliffe M., Huerta-Espino J., Lillemo M., Viccars L., Milne R., Periyannan S. (2015). A recently evolved hexose transporter variant confers resistance to multiple pathogens in wheat. Nat. Genet..

[19] Wang S., Basten C.J., Zeng Z.B. (2012). Windows QTL Cartographer 2.5.

[20] Voorrips R.E. (2002). MapChart: Software for the graphical presentation of linkage maps and QTL. J. Heredit..

[21] Nsabiyera V., Qureshi N., Bariana H.S., Wong D., Forrest K., Hayden M., Bansal U. (2016). Molecular markers for adult plant leaf rust resistance gene *Lr48* in wheat. Mol. Breed..

[22] Bariana H.S., Babu P., Forrest K.L., Park R.F., Bansal U.K. (2022). Discovery of the new leaf rust resistance gene *Lr82* in wheat: Molecular Mapping and Marker Development. Genes.

[23] Kumar K., Jan I., Saripalli G., Sharma P.K., Mir R.R., Balyan H.S., Gupta P.K. (2022). An update on resistance genes and their use in development of leaf rust resistant cultivars in wheat. Front. Genet..

[24] Ren X., Wang C., Ren Z., Wang J., Zhang P., Zhao S., Li M., Yuan M., Yu X., Li Z. (2023). Genetics of Resistance to Leaf Rust in Wheat: An Overview in a Genome-Wide Level. Sustainability.

[25] Kthiri D., Loladze A., N’Diaye A., Nilsen K.T., Walkowiak S., Dreisigacker S., Ammar K., Pozniak C.J. (2019). Mapping of genetic loci conferring resistance to leaf rust from three globally resistant durum wheat sources. Front. Plant Sci..

[26] Rosewarne G., Singh R., Huerta-Espino J., Rebetzke G. (2008). Quantitative trait loci for slow-rusting resistance in wheat to leaf rust and stripe rust identified with multi-environment analysis. Theor. Appl. Genet..

[27] Zhou Y., Ren Y., Lillemo M., Yao Z., Zhang P., Xia X., He Z., Li Z., Liu D. (2014). QTL mapping of adult-plant resistance to leaf rust in a RIL population derived from a cross of wheat cultivars Shanghai 3/Catbird and Naxos. Theor. Appl. Genet..

[28] Buerstmayr M., Matiasch L., Mascher F., Vida G., Ittu M., Robert O., Holdgate S., Flath K., Neumayer A., Buerstmayr H. (2014). Mapping of quantitative adult plant field resistance to leaf rust and stripe rust in two European winter wheat populations reveals co-location of three QTL conferring resistance to both rust pathogens. Theor. Appl. Genet..

[29] Iqbal M., Semagn K., Jarquin D., Randhawa H., McCallum B.D., Howard R., Aboukhaddour R., Ciechanowska I., Strenzke K., Crossa J. (2022). Identification of Disease Resistance Parents and Genome-Wide Association Mapping of Resistance in Spring Wheat. Plants.

[30] Leonova I.N., Skolotneva E.S., Salina E.A. (2020). Genome-wide association study of leaf rust resistance in Russian spring wheat varieties. BMC Plant Biol..

[31] Pasam R.K., Bansal U., Daetwyler H.D., Forrest K.L., Wong D., Petkowski J., Willey N., Randhawa M., Chhetri M., Miah H. (2017). Detection and validation of genomic regions associated with resistance to rust diseases in a worldwide hexaploid wheat landrace collection using BayesR and mixed linear model approaches. Theor. Appl. Genet..

[32] Sapkota S., Hao Y., Johnson J., Buck J., Aoun M., Mergoum M. (2019). Genome-wide association study of a worldwide collection of wheat genotypes reveals novel quantitative trait loci for leaf rust resistance. Plant Genome.

[33] Rosa S.B., McCallum B., Brûlé-Babel A., Hiebert C., Shorter S., Randhawa H.S., Barcellos A.L. (2016). Inheritance of leaf rust and stripe rust resistance in the Brazilian wheat cultivar ‘Toropi’. Plant Dis..

[34] Herrera-Foessel S.A., Singh R.P., Huerta-Espino J., Rosewarne G.M., Periyannan S.K., Viccars L., Calvo-Salazar V., Lan C., Lagudah E.S. (2012). *Lr68*: A new gene conferring slow rusting resistance to leaf rust in wheat. Theor. Appl. Genet..

[35] Zhang P., Li X., Gebrewahid T., Liu H., Xia X., He Z., Li Z., Liu D. (2019). QTL mapping of adult plant resistance to leaf and stripe rust in wheat cross SW8588/Thatcher using the wheat 55K SNP array. Plant Dis..

[36] Bokore F.E., Knox R.E., Hiebert C.W., Cuthbert R.D., DePauw R.M., Meyer B., N’Diaye A., Pozniak C.J., McCallum B.D. (2022). A combination of leaf rust resistance genes; including Lr34 and Lr46; is the key to the durable resistance of the Canadian wheat cultivar Carberry. Front. Plant Sci..

